# Automatic GAN-based MRI volume synthesis from US volumes: a proof of concept investigation

**DOI:** 10.1038/s41598-023-48595-3

**Published:** 2023-12-07

**Authors:** Damjan Vukovic, Igor Ruvinov, Maria Antico, Marian Steffens, Davide Fontanarosa

**Affiliations:** 1 School of Clinical Sciences, Queensland University of Technology, Gardens Point Campus, 2 George St, Brisbane, QLD 4000 Australia; 2 Centre for Biomedical Technologies (CBT), Queensland University of Technology, Brisbane, QLD 4000 Australia; 3 CSIRO Health and Biosecurity, The Australian eHealth Research Centre, Herston, QLD 4029 Australia

**Keywords:** Biotechnology, Anatomy, Health care, Mathematics and computing

## Abstract

Usually, a baseline image, either through magnetic resonance imaging (MRI) or computed tomography (CT), is captured as a reference before medical procedures such as respiratory interventions like Thoracentesis. In these procedures, ultrasound (US) imaging is often employed for guiding needle placement during Thoracentesis or providing image guidance in MISS procedures within the thoracic region. Following the procedure, a post-procedure image is acquired to monitor and evaluate the patient’s progress. Currently, there are no real-time guidance and tracking capabilities that allow a surgeon to perform their procedure using the familiarity of the reference imaging modality. In this work, we propose a real-time volumetric indirect registration using a deep learning approach where the fusion of multi-imaging modalities will allow for guidance and tracking of surgical procedures using US while displaying the resultant changes in a clinically friendly reference imaging modality (MRI). The deep learning method employs a series of generative adversarial networks (GANs), specifically CycleGAN, to conduct an unsupervised image-to-image translation. This process produces spatially aligned US and MRI volumes corresponding to their respective input volumes (MRI and US) of the thoracic spine anatomical region. In this preliminary proof-of-concept study, the focus was on the T9 vertebrae. A clinical expert performs anatomical validation of randomly selected real and generated volumes of the T9 thoracic vertebrae and gives a score of 0 (conclusive anatomical structures present) or 1 (inconclusive anatomical structures present) to each volume to check if the volumes are anatomically accurate. The Dice and Overlap metrics show how accurate the shape of T9 is when compared to real volumes and how consistent the shape of T9 is when compared to other generated volumes. The average Dice, Overlap and Accuracy to clearly label all the anatomical structures of the T9 vertebrae are approximately 80% across the board.

## Introduction

In image guided treatments often images taken at treatment stage are compared to pre-operative reference images, for example from CT or MRI. This enables surgeons to conduct minimally invasive and safer surgical procedures by relying on the visual information provided by the imaging modality used to capture the reference volume. This approach is applied in various procedures, including MISS^1^ in the thoracic region, as well as other respiratory interventions like Thoracentesis^2^.

Respiratory surgical procedures present a challenge in terms of image guidance due to the absence of real-time spatial information that can be linked to a pre-operative reference volume and the lack of a standardized imaging protocol to acquire the tracked volume. Current image guidance techniques rely on externally tracked imaging, such as infrared location tracking using a robotic arm^3^, or intraoperative imaging^4,5^ to provide a spatial link between the surgical equipment or imaging modality and the reference volume. This method utilizes spatial information provided by the external tracking system and is employed in automated image registration procedures to fuse the tracked volume with the reference volume. When applied to the challenges of volumetric pulmonary or spinal surgical image guidance, this method falls short. This limitation arises from its dependence on tracking-based deformation of a reference volume, which lacks the necessary accuracy when confronted with the intricate scene changes typically encountered in pulmonary or spinal surgical operations.

Image registration involves aligning two volumes or images of the same anatomical region by identifying corresponding landmarks within each volume. These landmarks are used to overlap the volumes, resulting in a single registered volume. Conventionally, during the process of registering two volumes, one is typically considered as the stationary or fixed volume (target), while the other volume (source) undergoes diverse image-based transformations to ensure alignment with the fixed volume. These transformations are applied to make the anatomical features within the two volumes coincide. However, in contrast to these direct image registration methods, there exists an alternative approach known as indirect methods or image-to-image translation methods. Within this framework, the algorithm acquires an understanding of the unique anatomical features and attributes that distinguish each imaging modality. It leverages this acquired knowledge to generate volumes that embody the traits of the alternate modality, and vice versa.

Indirect image registration through volume generation allows for the real-time volumetric tracking of US to be combined with the more interpretable MRI and CT imaging modalities. This enables the US imaging system to perform the guidance process and the MRI/CT imaging system to act as the visual aid during surgical procedures, since these modalities are the current gold standard for reference images of musculoskeletal and respiratory surgical procedures.

Registration of any volumes, whether between the same imaging modality (mono-modal) or different imaging modalities (multi-modal), can be done manually. The challenge is that traditional manual and automatic computer-aided approaches are incompatible to perform registration in real-time when complex elastic/diffeomorphic transformations are present. These complex transformations appear during the progression of an illness/disease/pathology. This problem can be alleviated using a deep learning (DL) approach to perform automatic real-time volumetric US/MRI image registration.

In this work, we propose an indirect automated image registration method using an unsupervised DL algorithm that synthesizes MRI volumes from a corresponding US volume. This method provides the surgeon with a spatially aligned MRI volume in real-time by training a DL algorithm on MRI and US volumes of the thoracic spine. The spatial congruence of the thoracic spine can facilitate additional alignment with the surrounding rib cage and pulmonary region. Given that these regions are interconnected and encase the lungs and heart, extending the spatial alignment of thoracic vertebrae with MRI synthesized by US volumes can involve incorporating real-time US volumes obtained from the ribs and other relevant anatomical regions. By employing this approach, we generate several spatially aligned US volumes using anatomical landmarks from the thoracic vertebrae and ribs. This enables us to effectively visualize and monitor pathologies within this region, encompassing areas such as the lungs and heart. Ultimately, these US volumes can be displayed in their respective MRI view, which offers a more familiar imaging modality for surgeons, making it easier for them to interpret and visualize these anatomical structures.

we present a DL approach aimed at translating US volumes to MRI volumes, facilitating real-time indirect registration of these two modalities. Our contribution can be summarized as follows:

1. A proof of concept showcasing the practicality of real-time volumetric indirect registration through phantom data of the thoracic vertebrae between 3D US and 3D MRI.

### Related works

The related works in multi-modal volumetric registration include US/MRI^6–9^, MRI/MRI^10–13^, MRI/Other^14,15^ and US or MRI based tracking/navigation^16,17^. Kaneko et al.^6^ implemented a supervised DL convolutional neural network (CNN) approach that is trained on prostate 3D MRI and US volumes that have been fused/registered beforehand using the Trinity workstation (Trinity®; Koelis, La Tronche, France). The 3D US data provided path data during the biopsy to determine the spatial location in the prostate in real-time.

In contrast, Haskins et al.^7^ manually registered MR-TRUS images of the prostate and used this dataset to train a DL algorithm to learn a new similarity metric. This similarity measure is classified as a regression problem, where the DL algorithm minimizes the target registration error between the registered pairs during image registration. Fu et al.^8^ performed automatic registration of MR and transrectal ultrasound (TRUS) images of the prostate to improve prostate cancer detection. This automated method uses deep learning-based 3D point cloud matching to overcome the challenges of non-rigid and deformable prostate tissue. The proposed method is biomechanically constrained to ensure accurate registration and improve the accuracy of prostate cancer detection. Jiao et al.^9^ Proposed a method for generating MR images of the brain (fetal) using US images as input, without the need for labelled MRI data. This self-supervised approach learns to generate MR images by predicting the missing US data and optimizing the network using adversarial and reconstruction losses of a monodirectional CycleGAN structure (US$\rightarrow$MR$\rightarrow$US). The results indicate that the proposed method outperforms existing methods in terms of image quality and similarity to real MRI images.

## Materials and methods

The dataset consists of one MRI volume and 220 US volumes of a thoracic spine phantom (including vertebrae and spinal processes). The MRI volume captures the whole thoracic spine region (T1–T12 vertebrae) and the US volumes capture the T9 spinal processes with slight shifts at each acquisition to include partial views of T8 or T10.

### Dataset

**Figure 1:**
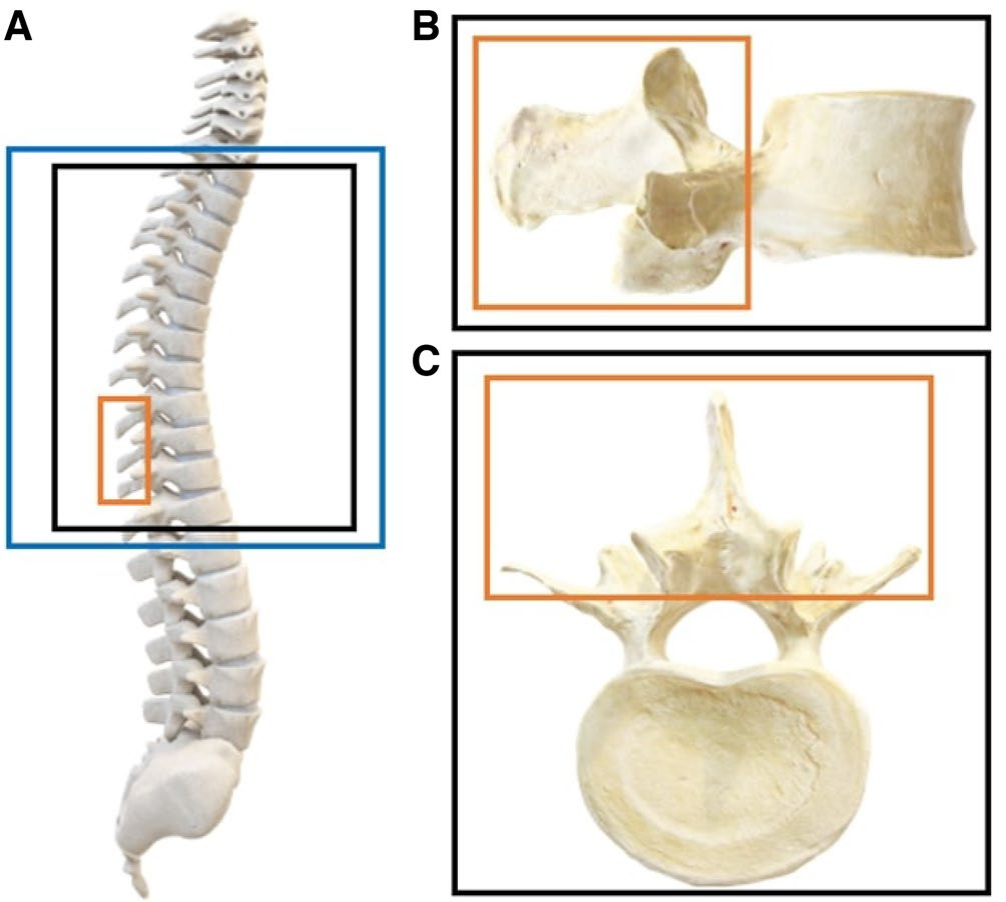
A sagittal view of an anatomical spine model (**A**) showing the thoracic spine region (blue outline) along with the MRI (black outline) and US (orange outline) capture volumes. The sagittal (**B**) and transverse (**C**) views of a single vertebra are also displayed with their respective MRI and US capture volumes.

**Figure 2:**
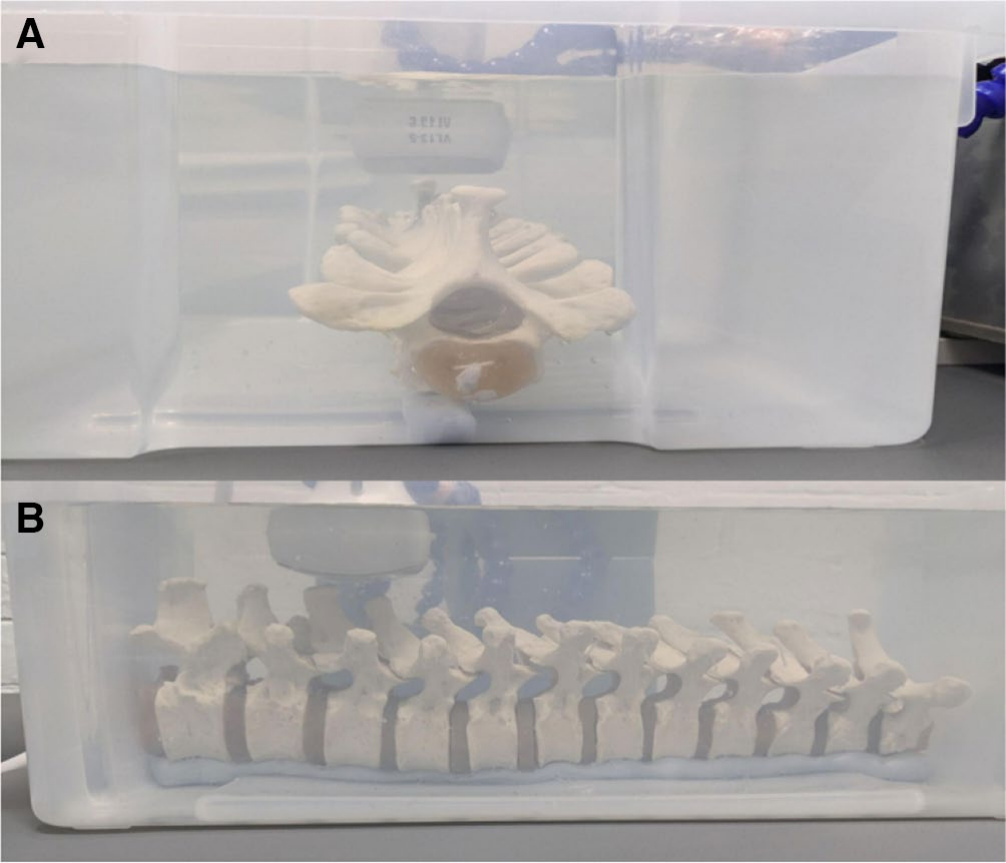
(**A**) presents a transverse view of the thoracic spine phantom, beginning from T1, while (**B**) showcases the corresponding sagittal view. The phantom is immersed in water, and the accompanying US probe used for capturing the respective volumes is also depicted.

Figure 1 displays the MRI (black outline) and US volume (orange outline) capture range during volume acquisition of the spine phantom. The acquisition of volume data involved a spinal model, as illustrated in Fig. 2, which was submerged in water. For the MRI scan^18^, a Siemens Magnetom Prisma 3T MRI system was employed to produce T2-weighted images (TR: 3200 ms; TE: 410 ms) with an isotropic voxel resolution of x = y = z = 0.9 mm. Throughout the MRI scan, the phantom spine model was maintained in a straight posture, ensuring alignment of the vertebral levels. In the resulting MRI images, the bony structure of the spine appeared as dark pixel intensities, while water was depicted as light pixel intensities.

The acquisition of the US volumes was carried out using a Phillips^TM^ VL13-5 broadband linear volume array US transducer, integrated with the Philips^TM^ Epiq 7G machine equipped with MaxVUe and xMatrix technology (frequency range 13–5 MHz, # of elements: 192, volume field of view (FOV): 38 mm $\times$ 30$^{\circ }$, modes: 2D/3D/4D, steerable pulsed wave and color Doppler, CPA, SonoCT, XRES, and multivariate harmonic imaging). The acquisition of US volumes involved submerging a spine phantom in water and manually conducting multiple scans at different positions and angles along the posterior thoracic spine. It’s important to note that no tracking system was implemented during the US volume acquisition process.

While Fig. 1A portrays the entire spinal region, encompassing the cervical, thoracic, and lumbar spine, our study exclusively targeted the thoracic (T1–T12) region, particularly T9, which is well-represented in the imaging modalities’ volumes. This emphasis on the thoracic spine is distinctly illustrated by the black rectangle within the anatomical model in Fig. 1A and in the live experimental setup presented in Fig. 2.

**Figure 3:**
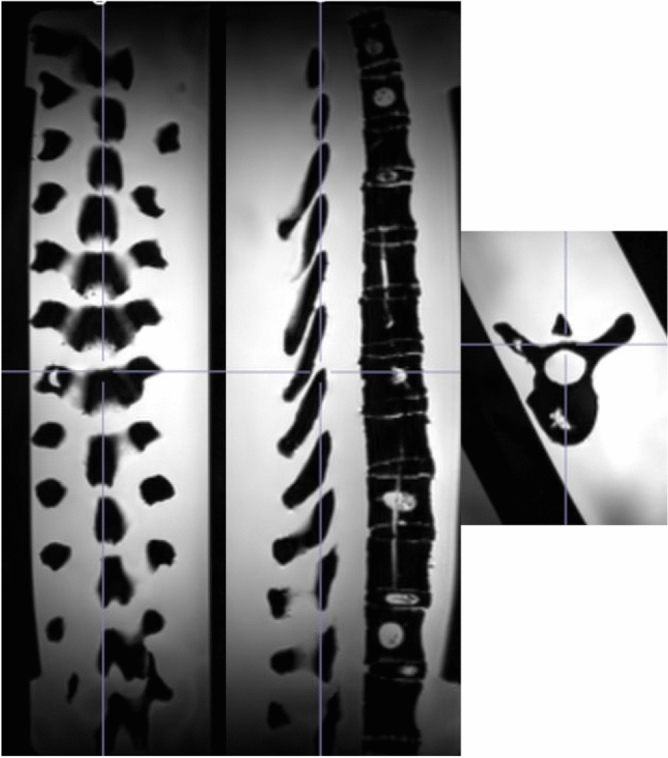
The original MRI volume of the thoracic spine region shown in the posterior coronal, sagittal and transverse planes (left to right).

As depicted in Fig. 1 and later in Figs. 3 and 5, the MRI volume comprises all the 12 thoracic vertebrae, while the US volume contains a complete view of one vertebra (T9) and two partial views of other vertebrae (T8, T10). Before being used as inputs into the training algorithm, these initial volumes will undergo processing.

**Figure 4:**
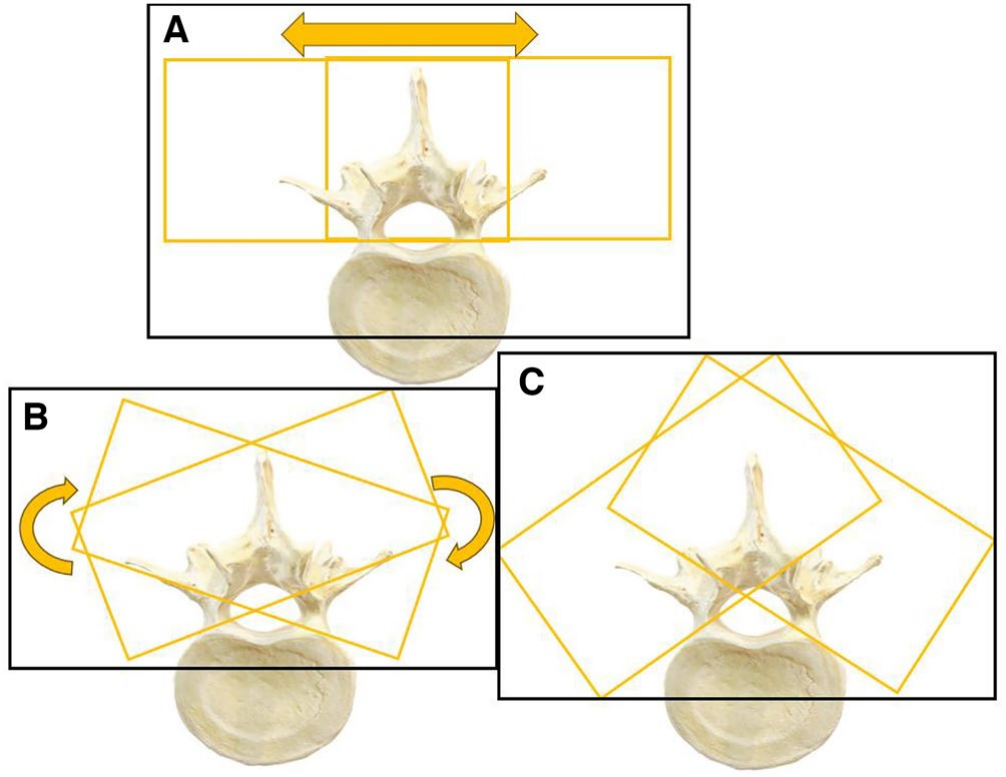
The US volumes were employed to train and assess the robustness and adaptability of the hyperparameters used in the algorithm. This dataset comprises US probe translations, as depicted in image A, as well as variations in US probe angles, as seen in images B and C.

To train and assess the algorithm’s hyperparameters, two distinct datasets were utilized: a training dataset and a test dataset. These datasets were distinct in that the test dataset included 20 previously unseen physical US volumes, exclusively used for testing purposes. These volumes, as depicted in Fig. 4, depict US scans (highlighted in orange) capturing the spinous and transverse processes of the thoracic vertebrae. A variety of combinations of these US volumes were recorded, following the US transducer angles and translation patterns delineated in Fig. 4.

**Figure 5:**
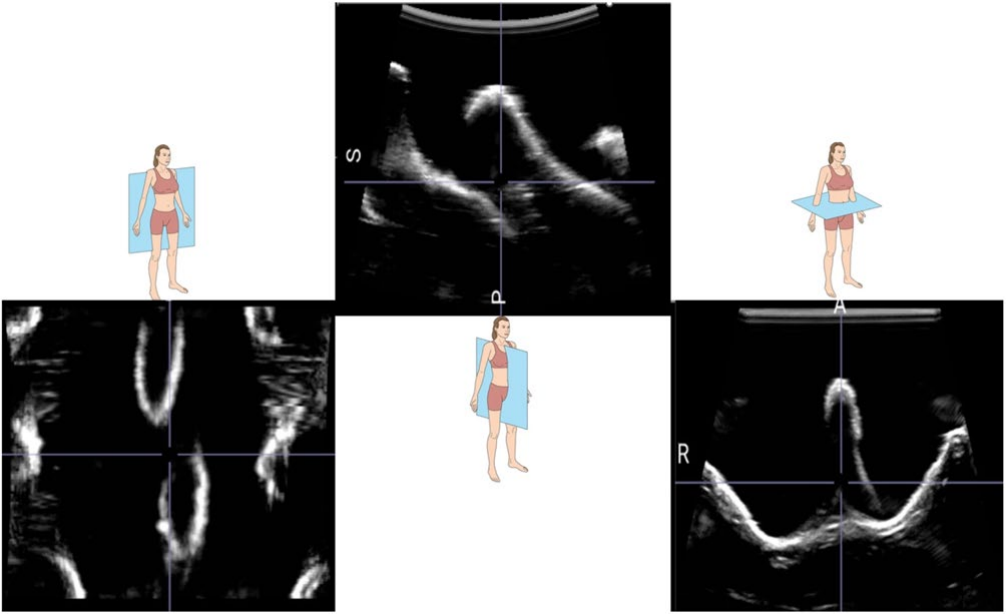
The original US volume of the thoracic spine region shown in the posterior coronal, sagittal and transverse planes respectively.

### Pre-processing

**Figure 6:**
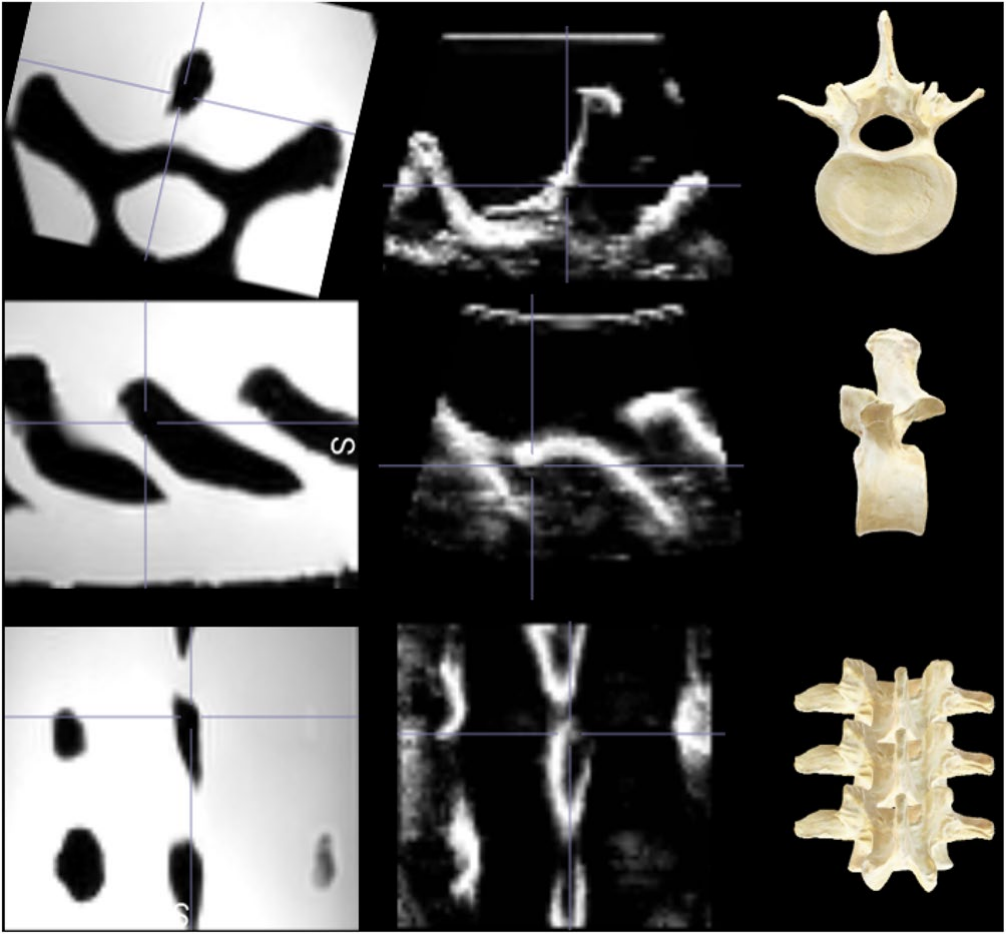
The transverse (top row), sagittal (middle row), and coronal (bottom row) views are shown of the processed US and MRI volumes used for training the algorithm. Each column from left to right shows the MRI, US, and respective anatomical model views. The US and MRI volumes were used to train the GAN algorithm. The anatomical models shown are not representative of the T9 vertebra, instead they are used as visual indicators of the transverse, sagittal, and posterior coronal views.

The original US voxel spacing/size is (0.129, 0.106, 0.250) mm as shown in Fig. 5 and its respective image size is (512, 451, 256) voxels along transverse, coronal, and sagittal planes, respectively. The voxel spacing of the US volume is adjusted through bilinear interpolation to match the MRI voxel spacing, ensuring a consistent aspect ratio (voxel size) as the MRI volume. This process results in a new US volume image size of (75, 53, 72) voxels, subsequently padded with black voxels uniformly around the entire volume. As a result, a new US volume image size of (96, 96, 96) voxels is achieved, as depicted in Fig. 6.

The original MRI (Fig. 3) voxel spacing/size is (0.885, 0.900, 0.885) mm and its respective image size is (270, 128, 384) voxels along transverse, coronal, and sagittal planes, respectively. The original MRI volume contains the thoracic spine region from T1–T12 vertebrae. This volume is cropped to a new size of (60, 85, 96) voxels to capture the field-of-view that is consistent with the US field of view (T8, T9, and T10) and is finally padded to (96, 96, 96) voxels (Fig. 6 first column).

### Deep learning model

This DL architecture maps one image domain to another using two sets of generative adversarial networks (GAN) that work together in an architecture known as CycleGAN^19^.

**Figure 7:**
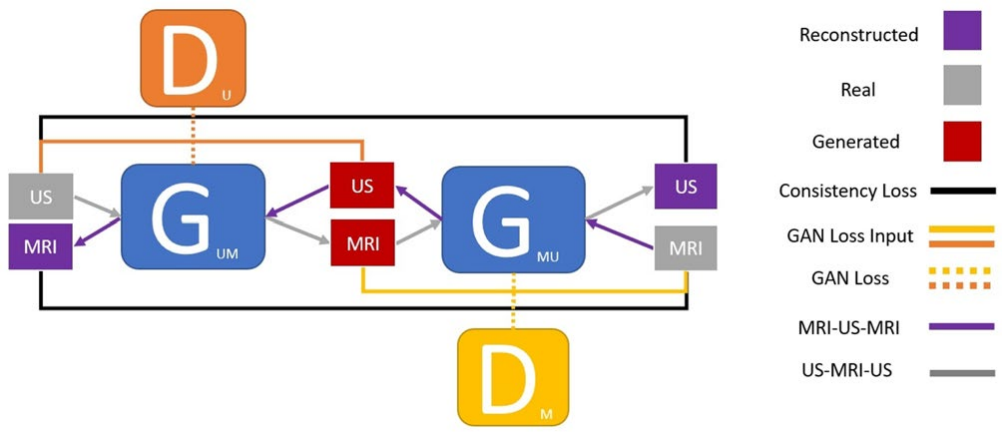
The general structure of CycleGAN and how the input images (US/MRI) are feed into their respective generators ($G_{UM}$ and $G_{MU}$) and discriminators ($D_{M}$ and $D_{U}$).

CycleGAN, as described in the work by Zhu et al.^19^, is a specific implementation of generative adversarial networks tailored for image-to-image translation tasks. CycleGAN aims to create a learned connection between two distinct domains, facilitating the smooth transition of images from one domain to another. One notable advantage of CycleGAN’s approach is that it can achieve this objective without the requirement for input data to be paired during the training phase. This achievement is facilitated by the integration of a cycle-consistency loss mechanism, ensuring that the conversion of an image from one domain to the other and its subsequent reverse translation maintain the fidelity of the original image.

Traditionally, image-to-image translation tasks have predominantly employed 2D CNN architectures, as the input data primarily comprises 2D images. However, in this research, the utilization of 3D volumes necessitated a slight modification in the architecture of the CNNs employed in CycleGAN. While maintaining a similar overall structure, these CNNs were adapted to use 3D convolutions instead of 2D convolutions, enabling the algorithm to effectively process and manipulate 3D volumes as input data.

The structure of the generator is based on an encoder/decoder with a U-net structure consisting of a Resnet 9 block backbone with 72 layers, where the discriminator structure consists of a decoder, specifically a PatchGAN^19^ discriminator, and a batch size of 2. The least squares loss is used for the GAN loss consisting of (shown in Fig. 7) $G_{UM}$/$D_{U}$, $G_{MU}$/$D_{M}$ generator/discriminator pairs. For the cycle consistency and identity loss, the sum of the L1 distance is used.

### Training approach

CycleGAN^19^ uses unpaired images during training of two generator/discriminator sets. Thus, the input MRI and US volumes do not need to be spatially aligned beforehand. Affine transformations are applied to the US and MRI volumes as part of the data augmentation process along with physical data augmentation before they are used as input into their respective generators. During each training epoch, the generators are fed with real US and MRI volumes with data augmentation applied (random XYZ rotation) and generate a new volume of the opposite imaging modality. The newly generated volumes are stored in a volume pool to provide the discriminator with historical data. The discriminators, on the other hand, evaluate the quality of either the newly generated volumes or previously generated volumes from the volume pool by comparing them with the corresponding real volumes.

The Weights and Biases^20^ framework was used to perform hyperparameter tuning using Bayesian optimization^21^ based on the performance of the US and MRI generated volumes, the training losses based on the cycle consistency loss, and the identity losses.

During training, US and MRI volumes are input into their respective generators ($G_{UM}$ and $G_{MU}$) and generate MRI and US volumes. The cycle consistency loss assesses the spatial alignment of the generated volumes regarding the original input volume in their respective discriminator. To verify this alignment, the generated volume is entered into the opposite generator to reconstruct a volume that represents the initial input volume. As the cycle loss decreases, the reconstructed volumes become indistinguishable from the original input, in turn reproducing the original volume. Therefore, the decrease in cycle loss indicates that the generated volume used to create the reconstructed volume is also spatially aligned with the original input volume. In summary, the cycle loss measures the similarity between the reconstructed volume and the original input volume, and thus, reflects the degree of spatial alignment between the generated volumes and the original input volumes.

During the training process, the identity loss is used to ensure that inputting a volume corresponding to a generator’s output modality into the generator results in an output volume that is identical to the input. For example, inputting an MRI volume into the generator which generates MRI volumes from US ($G_{UM}$) should output the original MRI volume. This ensures that the input volumes are not part of the image domain that the Generators are attempting to generate using the corresponding discriminator ($D_{M}$ for MRI and $D_{U}$ for US). As the identity loss decreases, $G_{UM}$/$D_{U}$ and $G_{MU}$/$D_{M}$ will respectively generate the input MRI and US volumes, also known as the identity volumes.

When training the modified CycleGAN algorithm, the primary focus was on the quality and visual appearance of the generated volumes. The generated volumes dictated which parameters needed to be adjusted during training, while the GAN losses were used to monitor and ensure that the progress of the generated images is progressing in the correct direction and that the algorithm is functioning effectively.

#### Training parameters

During training, the generator ($G_{UM}$ or $G_{MU}$) learns from the volumes in the training set and saves the generated volumes for each epoch in the volume pool. At the start of the run, newly generated volumes are added to the pool until it reaches its maximum capacity, which is a user-defined hyperparameter (Table 1). These generated volumes update the discriminator using a history of generated images^19,22^.

**Table 1:** Training hyperparameters: the model was trained using 100 volumes, and its performance was assessed on an additional set (validation set) of 100 volumes.

# Volumes	*200 US, 25 MRI*
Generator	*ResNet 9 block*
Generator learning rate	*0.0001*
Discriminator	*PatchGAN*
Discriminator learning rate	*0.0002*
Dropout	*0.8*
Pool size	*200*
Training time	*90 h*
Epoch	*250 static learning rate* + *250 linear decay learning rate* = 500 total

This evaluation facilitated the observation and refinement of parameters throughout the training process, resulting in the achievement of optimal hyperparameters prior to their implementation in the cross-validation stage.

The algorithm training was performed on a Linux workstation with an Intel i9-9820X CPU consisting of 20 cores running at 3.30 GHz, 2 Nvidia Titan RTX GPU with 24 GB of memory, and a total of 128GB of available memory. (Lambda Labs, San Francisco, CA, USA).

#### Cross validation/testing

A 5-fold cross validation was performed using the hyperparameters from Table 1. Table 2 shows the US volumes (labelled from 1 to 220 as subscripts) used in the training (T), validation (V), and testing (TE) sets.

The core training and validation datasets, labeled as “T” and “V” correspondingly, were employed for refining the model’s hyperparameters. During this iterative process, fine-tuning of the hyperparameters took place using the “T” dataset. These adjustments were driven by assessments of the training performance, which were guided by evaluations against the “V” dataset. Upon achieving optimized hyperparameters, their performance was subsequently validated using an unfamiliar test dataset identified as “TE”. This validation aimed to ensure the robustness and generalizability of the hyperparameters beyond the training data. The cross-validation strategy involved training the algorithm with the optimized hyperparameters using different combinations of the original “T” and “V” datasets, which have this new training dataset evaluated against the “TE” dataset. This dataset was not utilized in the initial training or validation stages, and encompassed new volumes captured through a diverse range of US probe placements and angles during the image acquisition process.

**Table 2:** The training (left), validation (right), and test (bottom) splits during the cross validation process.

Fold	Cross-validation	
1	$T_{21-100}$ , $V_{101-120}$	$T_{1-20}$ , $V_{121-200}$
2	$T_{1-20, 41-100}$ , $V_{121-140}$	$T_{21-40}$ , $V_{101-120, 141-200}$
3	$T_{1-40, 61-100}$ , $V_{141-160}$	$T_{41-60}$ , $V_{101-140, 161-200}$
4	$T_{1-60, 81-100}$ , $V_{161-180}$	$T_{61-80}$ , $V_{101-160, 181-200}$
5	$T_{1-80}$ , $V_{181-200}$	$T_{81-100}$ , $V_{101-180}$
	Test	
	$$TE_{201-220}$$	

### Evaluations

Before implementing the evaluation metrics, a preprocessing step was conducted to generate segmentation masks from the generated and actual volumes. This process involved cropping both types of volumes to include only the vertebrae, minimizing background presence. Following this, thresholding and grayscale value normalization were applied to accentuate the visibility of the bony anatomical structures (vertebrae). As a result, these processed volumes containing just the vertebrae were utilized to create thresholding masks, which were subsequently evaluated using the Dice and Overlap metrics.

#### T9 anatomical consistency

A quantitative assessment was carried out to evaluate the alignment of the T9 spinal and transverse processes within the generated MRI volumes, as well as between the real and generated volumes. This evaluation involved comparing the generated volumes with both real MRI volumes and additional generated MRI volumes. To achieve this, the Dice (Eq. 1)^23^ and Overlap (Eq. 2)^24^ metrics were employed. The main objective of these metrics was to examine the consistency of the T9 anatomical structure across the generated MRI volumes in comparison to the real MRI volumes. Through this analysis of T9 consistency, the aim was to determine the algorithm’s ability to consistently and accurately reproduce the distinct shape and anatomical characteristics associated with the T9 vertebrae.

1 $$\begin{aligned} Dice= & {} \frac{2 \left\| X \cap Y \right\| }{\left\| X \right\| + \left\| Y\right\| } = \frac{2 TP}{2 TP + FP + FN} \end{aligned}$$

2 $$\begin{aligned} Overlap= & {} \frac{\left\| X \cap Y \right\| }{min (\left\| X \right\| ,\left\| Y\right\| )} \end{aligned}$$

The Dice coefficient is computed as the intersection ($\cap$) between two volumes (X and Y) divided by the sum of the volumes, as illustrated in Eq. (1). When provided with a ground truth and a predicted value, Dice can be converted to use true positive (TP), false positive (FP), and false negative (FN) rates. The Overlap or Szymkiewicz-Simpson, as shown in Eq. (2), is similarly calculated by dividing the intersection of two volumes by the smaller volume, focusing on the common area that volumes X and Y overlap.

#### Clinical discriminator validation

Within the CycleGAN algorithm, the discriminators $D_{U}$ and $D_{M}$ evaluate the generated US and MRI volumes produced by $G_{MU}$ and $G_{UM}$, respectively, to ensure their alignment with their respective image domains. The generators are responsible for crafting volumes that showcase the spinal and transverse processes of the T9 vertebra, aiming to deceive the discriminator by generating images that closely resemble real volumes.

Additionally, a skilled clinician carried out a similar evaluation based on images, mirroring the role of GAN discriminators. This expert identified and labelled anatomical markers within a random set of real and generated MRI volumes, keeping these labels undisclosed during the process. The clinician assigned binary qualitative scores based on their ability to differentiate the transverse and spinal processes of the generated T9 vertebrae. A score of 0 indicated a distinct separation between the anatomical structures within the generated T9 vertebrae, while a score of 1 indicated a lack of clarity in anatomical markers. This evaluation was conducted on 20 generated volumes.

The US and their generated MRI volume counterparts had a registration evaluated by the expert. This was done by taking each volume and orientating it such that the spinous process was directed and pointed up on the screen, where then the most superficial part of the spinous process was labelled for both MRI and US volumes. Once this was completed, the volumes with this anatomical label were opened using another software where the target registration error (TRE)^25^ can be measured between the spinous process label for the US and MRI volume given as a quantitative value in mm.

**Figure 8:**
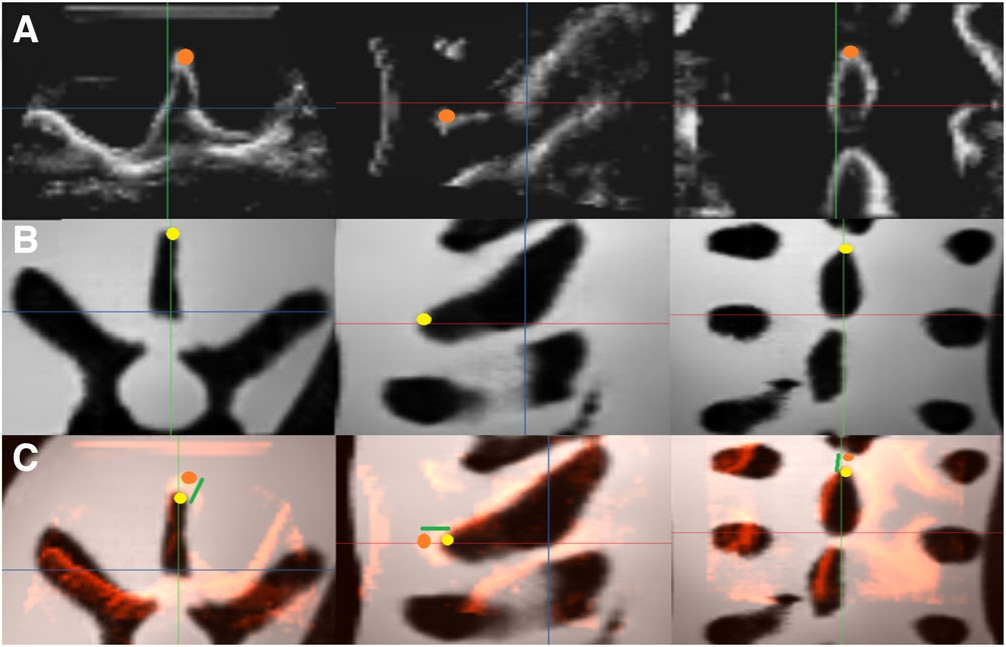
The clinical expert assesses the algorithm’s volume registration accuracy by qualitatively analyzing the spatial alignment between the input US volume and its corresponding generated MRI volume (row C, green line segment) using the target registration error. This evaluation offers additional quantitative insights, focusing on the anatomical characteristics of the thoracic vertebrae present in both volumes. The images depict the input US volume (row A, orange dot) and its corresponding generated MRI volume (row B, yellow dot) along the transverse, sagittal, and coronal planes, arranged from left to right.

Additionally, the expert conducted a qualitative evaluation to gauge the alignment or registration quality between the input US volume and the generated MRI volume. This assessment was grounded in clinical evaluations of essential anatomical structures within the thoracic vertebrae, including the transverse and spinous processes, and is shown in Fig. 8.

Ultimately, a quantitative analysis was performed to assess the distinction between the test and training sets. This evaluation included the use of the Target Registration Error (TRE) metric, which gauges the variation between the 100 training US volumes and the 20 test US volumes. This difference is visually represented in Fig. 4. Additionally, a quantitative assessment was conducted to objectively compare the training and test datasets. This was achieved by employing the perceptual distance similarity metric, as outlined in^26,27^. This metric replicates the human ability to perceive changes through visual judgment, providing a reliable means of comparison between the two datasets.

## Results

**Figure 9:**
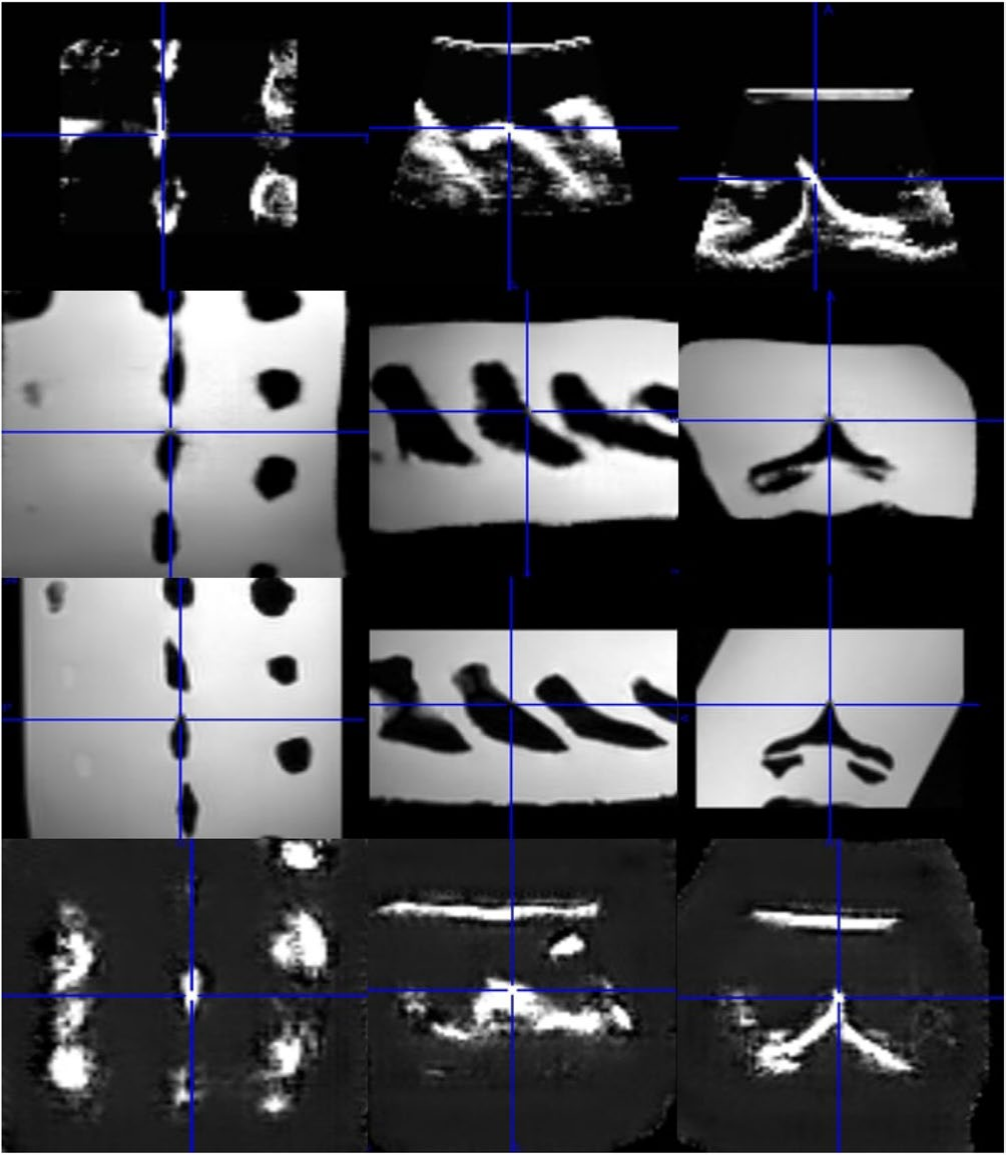
The rows (from top to bottom) represent the real US, generated MRI, real MRI, and reconstructed US respectively. The columns (from left to right) show the posterior coronal, sagittal, and transverse planes respectively.

The clinical validation performed by the expert resulted in an 80% anatomical validation, where the transverse and spinal processes of the generated volumes in the cross-validation folds were clearly visible and identifiable. The remaining 20% fall into the category of poor image quality that resulted in an inconclusive decision whether the spinal, transverse or both processes were clearly identifiable. A representation of the reconstructed US volume, alongside both the generated and real MRI/US volumes, are shown in Fig. 9.

**Table 3:** T9 Dice calculated between the generated volumes from the 5-fold cross validation and the real ground truth volumes.

Fold	Worst	Average	Best
1	0.774	0.792	0.816
2	0.761	0.780	0.799
3	0.780	0.800	0.819
4	0.790	0.808	0.828
5	0.772	0.791	0.806

**Table 4:** T9 Overlap calculated between the generated volumes from the 5-fold cross validation and the real ground truth volumes.

Fold	Worst	Average	Best
1	0.800	0.813	0.831
2	0.790	0.801	0.812
3	0.789	0.805	0.824
4	0.795	0.813	0.845
5	0.785	0.807	0.826

Tables 3 and  4 show the Dice and Overlap evaluation metrics calculated for the 5-fold cross validation between real and generated MRI volumes. The same evaluation was performed between generated MRI volumes, and their Dice and Overlap metrics are shown in Tables 5 and  6.

**Table 5:** T9 Dice calculated between the generated volumes from the 5-fold cross validation.

Fold	Worst	Average	Best
1	0.797	0.865	0.932
2	0.806	0.860	0.915
3	0.843	0.890	0.954
4	0.842	0.895	0.948
5	0.845	0.894	0.957

**Table 6:** T9 Overlap calculated between the generated volumes from the 5-fold cross validation.

Fold	Worst	Average	Best
1	0.798	0.873	0.945
2	0.806	0.870	0.925
3	0.845	0.898	0.961
4	0.854	0.903	0.953
5	0.853	0.899	0.959

The TRE evaluations on the test dataset, an average of 3.904 mm ± 0.579 and 8.31 mm ± 0.570 was obtained on the 20 US volumes in this set. The two TRE values resulted from the test dataset consisting of 10 US volumes that had complete views of the spinous and transverse processes with slight rotations (SR from Fig. 4, and 10 volumes with partial views of one of the two transverse processes (MR,P from Fig. 4).

**Table 7:** Perceptual distance calculated between the 100 training US volumes against the 20 US test volumes. Where the mild rotation (MR)/slight rotation (SR) of the ultrasound probe, and partial view of the thoracic vertebrae (P) is based on Fig. 4.

Perceptual distance	Training vs testing set	
	MR,P	SR
Lowest	0.3509	0.3471
Highest	0.3541	0.3589
Average	0.3530	0.3507

Where the perceptual distance (shown in Table 7 shows a significant difference between the training and test set along with a difference between the subsets (MR,P and the SR) of the test set. This is further validated by performing the perceptual distance between the US and MRI sets as shown in Fig. 8.

**Table 8:** Perceptual distance calculated between the US and MRI volumes.

Perceptual distance	US vs MRI	
	MR,P	SR
Lowest	0.5551	0.5512
Highest	0.5803	0.5855
Average	0.5630	0.5667

## Discussion

The primary objective of this preliminary proof-of-concept study was to demonstrate the capability and feasibility of achieving real-time volumetric translation from US to MRI (indirect registration) within the context of the bony thoracic anatomical structures. This serves as the foundational step for future endeavors, including the integration of actual patient data, to advance the envisioned clinical applications of this technique. A significant constraint in image-to-image translation tasks lies in the potential of the algorithm, during training, to produce volumes containing anatomical structures, and modality-specific features unique to MRI or US that do not exist or are duplicated from the training dataset. Addressing this challenge becomes crucial and necessitates meticulous consideration and integration during the training process. While demonstrating the viability of real-time volumetric translation from US to MRI and considering the evaluation metrics that confirm substantial differences between the test and training images, it’s essential to exercise caution. The results might be somewhat influenced by the possibility of test images overlapping or recurring during image acquisition, which could introduce some bias into the outcomes.

Generating content and style that may not naturally appear in the generated imaging modalities is attributed to discrepancies in style and content between the source and target imaging domains. This phenomenon arises due to an insufficiently diverse dataset that fails to encapsulate the distinct content and style attributes unique to each imaging domain. Consequently, CycleGAN employs techniques like overlapping the input MRI or US volume during the volume generation process. In these cases, the model tends to “cheat” by recreating images based on the overlapped volumes it has seen, neglecting the incorporation of both style and content information. This occurs because the model becomes overly accustomed to the training dataset it was exposed to, mimicking the style and content while essentially duplicating the same image. In reality, the model does not adequately consider the original input volume, resulting in a generated MRI volume, for instance, that portrays the features or style reminiscent of an MRI of the thoracic vertebrae but lacks alignment with the initial input volume. Consequently, the model fabricates features or style characteristics of MRI images, along with the content of bony structures, within these newly generated images.

To mitigate the phenomenon of ‘dreaming of content and style’, in this work a more precise adjustment (reduced range) of hyperparameters is necessary, alongside the inclusion of a substantial number of representative US and MRI volumes. In our methodology, we adopted a dataset of 100 US volumes and 25 MRI volumes as a minimum requirement to effectively address this issue, aiming to minimize the extensive time and computational resources typically demanded by the training process.

The integration of real-time anatomical spatial information can facilitate real-time pathology monitoring and accurate volume estimation, particularly when combined with an automated volumetric segmentation system. This work has the potential to enhance previous classification and segmentation work conducted on lung ultrasound (LUS) imaging for pleural effusion (PE).

The addition of volumetric data of the lung and thoracic spine to this work would enable indirect registration of these regions, including the spine, ribs, and lung, thereby enhancing spatial awareness during pathology monitoring or fluid estimation in the lungs. Once indirectly registered, the resulting volume can be displayed in the widely used imagining modality of MRI or CT. Finally, this work may serve as a foundation for future use in capacitive micromachined ultrasonic transducers (CMUT) 3D US imaging.

The image quality of the generated volumes served as a visual cue during training to implement a binary search optimization method for determining the optimal number of training iterations (epochs) elapsed before beginning to decay the learning rates linearly. Our training consisted of 250 epochs with a static learning rate, followed by an additional 250 epochs (total 500) with a linearly decaying learning rate. Multiple trade-offs were implemented to reduce computational resources and training time with minimal losses to image quality of the final generated volumes. The trade-offs included reduction of image size, number of training images, and number of layers for the generators, as well as maintaining volume aspect ratio between MRI/US. On average, over the 5 cross validation folds, the Dice and Overlap scores were both around 0.8, and there was an 80% rate of clear visibility for T9 anatomical structures when the expert validated the generated volumes for image quality. The other 20% of test cases showed partial visibility of either the transverse or spinal processes, or neither, due to poor image quality and artifacts. To improve this, increasing the number of layers in the generator could be considered to allow for higher quality input volumes during training. However, this would require significant additional computational resources and training time for relatively small improvements at present since the focus was on developing an algorithm for indirect registration of US and MRI volumes, by generating MRI volumes that are spatially aligned with the original US. Future work could include scaling up the algorithm and addressing other limitations when working with patient data.

Beyond the Dice and Overlap metrics, an important consideration was the performance of the generated MRI volumes when registered with their corresponding US volumes, which served as their source data. TRE was used to assess the registration portion. The TRE was computed by measuring the distance between anatomical markers, specifically the spinous process, which was consistently visible in the test dataset on both the US and MRI volumes. This analysis resulted in two separate TRE values: 3.904 mm ± 0.579 and 8.31 mm ± 0.570 for the complete and partial views of the transverse processes, respectively.

The necessity for two TRE values arose due to the composition of the test dataset. Half of the volumes in this dataset featured partial views of one of the transverse processes and were acquired using a larger/steeper angle (as seen in Fig. 4, image C). These factors presented challenges for the algorithm during registration, leading to relatively high TRE values. However, it is important to note that this limitation stems from the lack of additional volumes in the training dataset that encompassed these extreme US cases and performed rather well for the rest of the cases as an initial proof-of-concept work. In future endeavors, following the successful proof of concept presented here, we aim to leverage the insights gained from these metrics (Dice, Overlap, TRE) using real patient data and further refine the algorithm.

Incorporating the perceptual distance metric, as illustrated in Fig. 7 for the US training and test sets and Fig. 8 for the US and MRI sets, provided an effective measure for quantifying the disparities between the datasets due to variations in probe rotation and translation during image acquisition (refer to Fig. 4). These results clearly demonstrate that the testing volumes exhibit significant dissimilarities compared to the training volumes.

Regarding the choice of the least square loss for the GAN loss, this loss function was observed to be more stable and generate higher quality images compared to other loss functions. The least square loss function is a variant of the GAN loss function that minimizes the mean squared error between the generated and real images. This results in more stable training and better convergence of the generator network. Additionally, the least square loss function encourages the generator to produce more realistic and high-quality images.

The training of deep learning algorithms that rely on 3D input volumes become even more resource intensive as a larger dataset is needed and the algorithm architecture increases. To address this, potential algorithm efficiency improvements could be made including the use of transfer learning, transformers, or other deep learning approaches and optimization techniques.

This preliminary proof-of-concept study was carried out using phantom data. However, future phases of this research will require the incorporation of patient data from clinical studies, and therefore the inclusion of patient data was not within the scope of this initial pilot/proof-of-concept work. Furthermore, upcoming research will delve into the investigation of multi-scale resolution GANs to adapt to diverse input volume sizes and voxel spacing. The inclusion of additional optimization techniques will be introduced as a component of ongoing research endeavors.

While both MRI and CT scans provide insights into pathology, only US offers the ability to monitor the progression of pathology volumetrically in real time. This significance extends to not just pathology monitoring or other potential topographical anatomical changes, but also to the tracking of dynamic changes within the thoracic cavity, encompassing the movements of spinal vertebrae as an example. The algorithm’s training process can encompass the capability to differentiate between individual vertebrae, thereby facilitating the potential for automated registration between the generated MRI volume and the comprehensive MRI reference volume. This holds relevance in addressing the challenge of vertebral identification during surgery, where the user can view either through the lens of the US, generated MRI, or the entire reference MRI. By visualizing the US scan within the context of the complete MRI reference volume, the positioning of the probe becomes discernible, offering the user a visual cue of the currently observed vertebrae.

The algorithm capitalizes on the limited FOV inherent in the input US volume to create an MRI volume with spatial alignment, effectively establishing intrinsic registration between the two modalities. This is particularly significant because the generated MRI volume can subsequently be aligned with the comprehensive reference MRI volume, encompassing the entire area of interest, such as the thoracic spine. This goes beyond the constraints of single or limited vertebrae registration and enables US-to-MRI image guidance.

Consequently, the necessity for spatial tracking mechanisms, such as infrared tracking, for the input US volumes is eliminated. The algorithm performs the critical task of indirect registration through real-time volume-to-volume translation. This dynamic process allows for immediate reflection of changes in the US volume, such as tissue characterization, in the corresponding MRI volume. The registration procedure empowers users to precisely determine the spatial positioning of the generated MRI volume in relation to the entire reference MRI volume. This insight enables them to visualize the placement of the US probe during the initial volume acquisition within the context of the comprehensive thoracic spine, in contrast to the limited FOV of the original scan.

It’s important to note that this represents just one of the numerous potential applications stemming from this initial proof-of-concept endeavor.

In future work, the integration of actual patient data, diverse multi-resolution volumes, and datasets comprising both healthy and pathological cases is planned. This initial proof-of-concept study aimed to validate the feasibility of volume-to-volume translations between US and MRI, specifically to obtain a spatially aligned MRI volume corresponding to the input US volume of the thoracic vertebrae. The choice of phantom data for this study was primarily driven by the unavailability of volumetric patient data and the emphasis on establishing the core concept of volume translation. Prospective applications of this research may encompass minimally invasive spine surgeries, particularly procedures involving spinal pedicle screw placement. Furthermore, future expansion initiatives could involve conducting an ablation study. In this study, the complete thoracic region would be mapped in ultrasound concerning MRI, and these datasets could then be presented to sonographers for evaluation. Additionally, this comprehensively mapped US of the thoracic region which is aligned with reference volume (MRI) will allow for the automated detection and diagnosis of pathologies within the thoracic region. Expanding on the application of a comprehensive thoracic ultrasound map, thanks to the relatively brief algorithm training period, it becomes feasible to develop a dedicated model for each patient. This individualized model is constructed using the patient’s MRI and their pre-surgical ultrasound volume data. Subsequently, this personalized model can be deployed during the patient’s surgery.

## Conclusion

In summary, this study’s proof-of-concept for generating MRI and US volumes of the thoracic vertebrae is a promising first step towards providing surgeons with real-time MRI volume guidance during surgical procedures. Further research may investigate the potential of utilizing transformers or other deep learning methods to design a more powerful algorithm that can handle real patient volumes acquired via CMUTs or other volumetric US imaging techniques for the purposes of monitoring and diagnosing pathology throughout the entire thoracic anatomical area, with a particular emphasis on classification and segmentation tasks.

## Data Availability

The datasets used and/or analyzed during the current study are available from the corresponding author(s) on reasonable request.
